# ReadDepth: A Parallel R Package for Detecting Copy Number Alterations from Short Sequencing Reads

**DOI:** 10.1371/journal.pone.0016327

**Published:** 2011-01-31

**Authors:** Christopher A. Miller, Oliver Hampton, Cristian Coarfa, Aleksandar Milosavljevic

**Affiliations:** 1 Graduate Program in Structural and Computational Biology and Molecular Biophysics, Baylor College of Medicine, Houston, Texas, United States of America; 2 Department of Molecular and Human Genetics, Baylor College of Medicine, Houston, Texas, United States of America; University of Leuven, Belgium

## Abstract

Copy number alterations are important contributors to many genetic diseases, including cancer. We present the readDepth package for R, which can detect these aberrations by measuring the depth of coverage obtained by massively parallel sequencing of the genome. In addition to achieving higher accuracy than existing packages, our tool runs much faster by utilizing multi-core architectures to parallelize the processing of these large data sets. In contrast to other published methods, readDepth does not require the sequencing of a reference sample, and uses a robust statistical model that accounts for overdispersed data. It includes a method for effectively increasing the resolution obtained from low-coverage experiments by utilizing breakpoint information from paired end sequencing to do positional refinement. We also demonstrate a method for inferring copy number using reads generated by whole-genome bisulfite sequencing, thus enabling integrative study of epigenomic and copy number alterations. Finally, we apply this tool to two genomes, showing that it performs well on genomes sequenced to both low and high coverage. The readDepth package runs on Linux and MacOSX, is released under the Apache 2.0 license, and is available at http://code.google.com/p/readdepth/.

## Introduction

Copy number alterations (CNAs) that arise due to genomic duplications or deletions have gradually been recognized as a major contributor to genetic variation and disease [1]–[4]. In addition to being linked to Mendelian diseases, CNAs have been frequently described in tumor genomes and contribute to oncogenesis by altering gene dosage or creating gene fusions or truncations [5], [6]. In order to assess the effects of CNAs in either normal or tumor genomes, it is necessary to both precisely demarcate the boundaries of the alteration and accurately infer the copy number. In the past decade, this has typically been done using array comparative genomic hybridization (aCGH) [7], [8]. Despite the low cost and ubiquity of such methods, aCGH is beginning to be supplemented by sequencing-based methods. These methods offer a number of advantages, including higher resolution and better dynamic range [9]–[11].

Specifically, it has been shown that when whole-genome shotgun sequencing is performed on massively parallel instruments, the number of sequence reads that align to a position in the genome is proportional to the copy number at that position [9]. This simple concept is complicated by the fact that genomes are not sequenced deeply enough to enable base-pair resolution. This necessitates the use of a binning procedure, where bins of a fixed size are tiled along the genome and the reads falling into each bin are counted. The size of the bin and number of reads determine how accurately normal regions can be distinguished from those with amplifications or deletions [11], [12]. Thus, the statistical methods used to model the dataset and determine the size of these bins are a key component of an accurate detection algorithm. Some algorithms arbitrarily choose this bin size [9], [10], which is clearly less than optimal, while others use models based on Poisson or Gaussian distributions to determine parameters that result in good sensitivity and a small bin size which gives good resolution [11]–[13]. If the model's assumptions are violated and the bin size is too large or small, the algorithm's performance will suffer.

Another challenge in extracting copy number information from sequence data is that the genome contains many repetitive elements, and aligning reads to these positions is impossible using current short-read technologies. Several groups sidestep this problem by sequencing a reference genome alongside their target genome [11]–[13]. This allows the algorithm to use the ratio between the two samples at each position and reduces this problem of “mapability”. Unfortunately, this also effectively doubles the costs of sequencing, which is still a significant expense.

We present readDepth, a new R package for CNA detection that does not require the sequencing of matched normal sample. Using a binning procedure, readDepth calls copy number variants based on sequence depth, and then invokes a circular binary segmentation algorithm to call segment boundaries. If the reads are obtained from paired ends, breakpoint information can be used to refine segment boundaries. The algorithm accepts both regular and bisulfite-treated DNA reads, thus enabling integrative study of structural and epigenomic alterations. A key feature of the algorithm is an improved statistical model that is applied to adjust for a number of types of bias, including GC-content, mapability, and other sources of distortion introduced by the preparation and sequencing processes. The algorithm also allows for explicit control of the false discovery rate (FDR), which minimizes the number of false positive aberrations detected. Furthermore, by leveraging parallel processing, readDepth produces results many times faster than comparable algorithms.

We validate readDepth on simulated data and show that it has high sensitivity and specificity and outperforms a comparable R package. We then apply it to several genomes. We show this algorithm's ability to resolve complex and highly amplified regions from low-coverage data on the MCF-7 breast cancer cell line. We also demonstrate a method for integrating information from paired-end sequencing information to create more accurate boundary calls. Finally, we showcase a methylation-sensitive method of correcting for GC-content bias by applying it to high-coverage bisulfite-treated data from the H1 embryonic stem cell line.

## Results

### Statistical Model and Algorithm Development

We first examined several genomes and assessed the distribution of reads uniquely mapping to the genome in order to create a statistical model. We started with the assumption that all reads were chosen randomly from the genome, which meant that the number of reads in any given region would follow a Poisson distribution with mean proportional to the copy number of the region. After examining several complete genomes (Yoruban [14], Han Chinese [15], and Korean [16]), we noted that the observed distributions violate the Poisson distribution's assumption of equal mean and variance, even after correcting for several known sources of distortion such as GC-content bias and variation in mapability.

**Figure 1:**
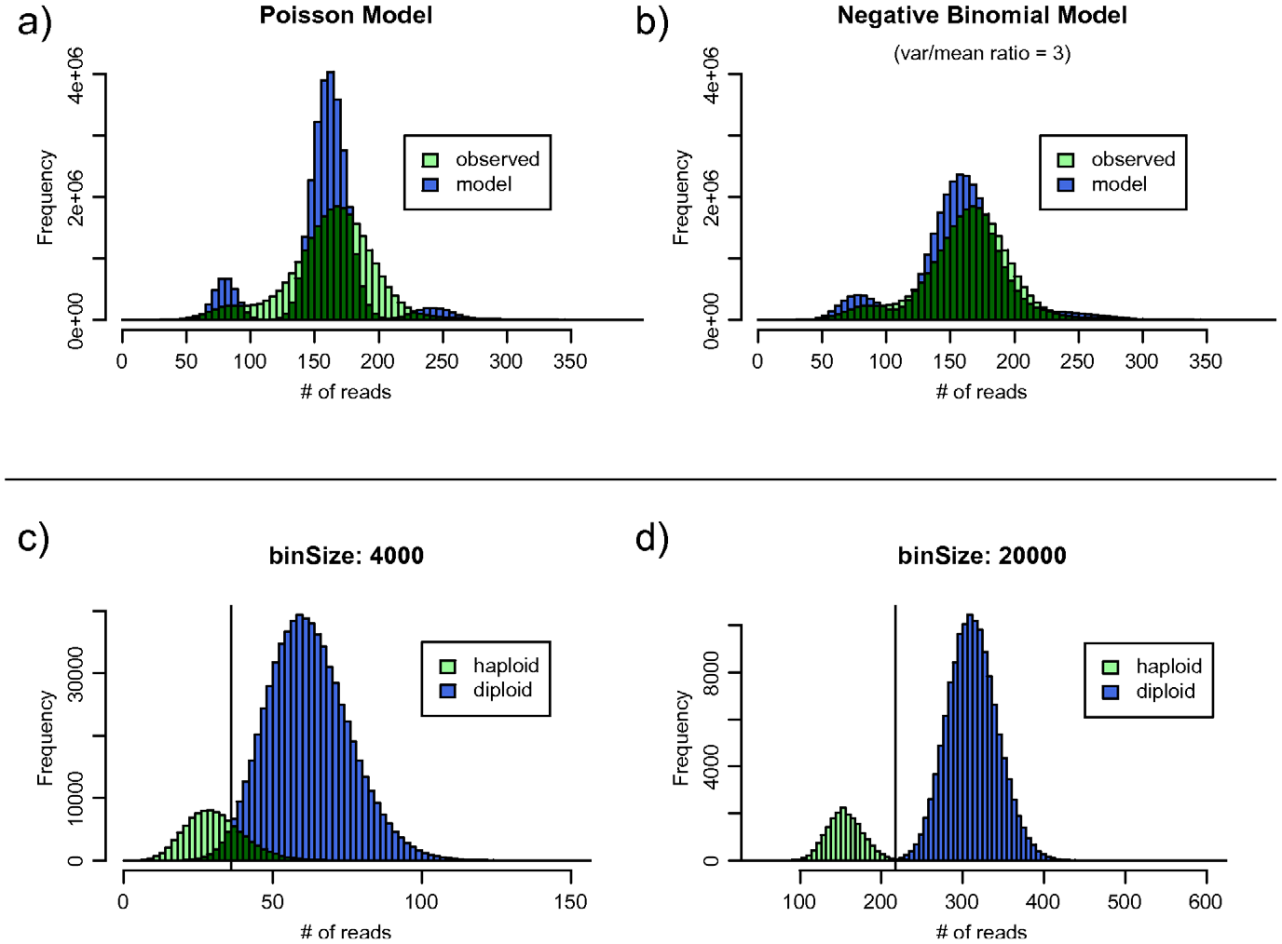
Bin size determination and distribution modeling. a) Illumina reads from the Yoruban genome are not fit well by a Poisson model. b) Modeling the reads using a negative binomial distribution with a variance/mean ratio of 3 results in a much better fit, with a root mean square error three times smaller. c) The use of bin sizes that are too small results in an inability to cleanly separate peaks with copy number of one and two, resulting in a large number of false-positive calls in the overlapping region. d) Increasing the bin size allows us to trade resolution for better separation.

Thus, we developed a model that uses a negative-binomial distribution to approximate an overdispersed Poisson distribution. The negative binomial distribution can be seen as a mixture of Poisson distributions where the median values (λ) are drawn from a gamma distribution [17]. The variation in λ, which accounts for the excessive variance which we observe, is input to the model using an extra parameter. By altering this parameter, we can alter the variance/mean ratio (VMR) and model overdispersion. A similar approach for modeling overdispersion has been proposed in the context of detecting gene expression levels from sequence reads [18], but has not previously been applied to copy number assays. We concluded that the genomes we examined, which were all sequenced on Illumina machines, are well approximated by a negative binomial distribution with a VMR of 3. On the Yoruban genome (visualized in Figure 1a/b), our negative binomial model has a root mean square error three times smaller than that of the Poisson (RMSE of 23817.203 vs 7955.012).

We next developed readDepth, a tool that uses our model to identify sets of optimal parameters that correspond to specific false-discovery rates. To allow for improvements in the sequencing process or introduction of new platforms that may result in different distributions, the VMR parameter (set to 3 in the following experiments) is adjustable. Figures 1c and 1d show the effects of different bin sizes, which control the mean number of reads per bin, and by extension, control the separability of peaks at each copy number. Given a data set with a certain number of reads, readDepth calculates the smallest bin size that allows no more miscalled bins than specified by the input FDR, then calculates the thresholds for copy number gain and loss that optimally separate the peaks.

Once a bin size is established and the number of reads that fall into each bin is counted, readDepth corrects for bias introduced by the inability to map reads into repetitive regions of the genome. To do this, we created mapability tracks via self-alignment of the reference genome. ReadDepth takes these tracks as input and uses them to proportionally scale the number of reads in bins that have less than 100% mapability. Previous studies have described significant bias in the number of reads generated from regions with differing GC-content [14]. Based on this observation, which we have confirmed, readDepth uses a simple statistical correction to adjust the read depth for this bias. (Figure S1)

Once read counts are adjusted for each bin in the genome, readDepth applies circular binary segmentation, as implemented in the DNAcopy R package [19], to divide the genome into contiguous regions with the same copy number. It then reports copy numbers for each segment, and flags segments that exceed the thresholds for gain and loss calculated by the model.

### Validation

We first validated readDepth's ability to detect regions of copy number gain and loss using simulated data. We randomly generated reads from chromosome one, drawing from a distribution with copy number of two that conforms to our negative-binomial model with a VMR of three. We then insert a copy number gain by replacing a region of the specified size with reads drawn from a distribution with a copy number of three. One thousand simulations were run, and sensitivity was measured by determining if both edges of the CNA call matched the seeded CNA with a tolerance of one bin size. A false positive was defined as a putative alteration call where both ends did not match the seeded CNA. We measured specificity by taking one minus the percentage of trials which had one or more false positive calls.

**Figure 2:**
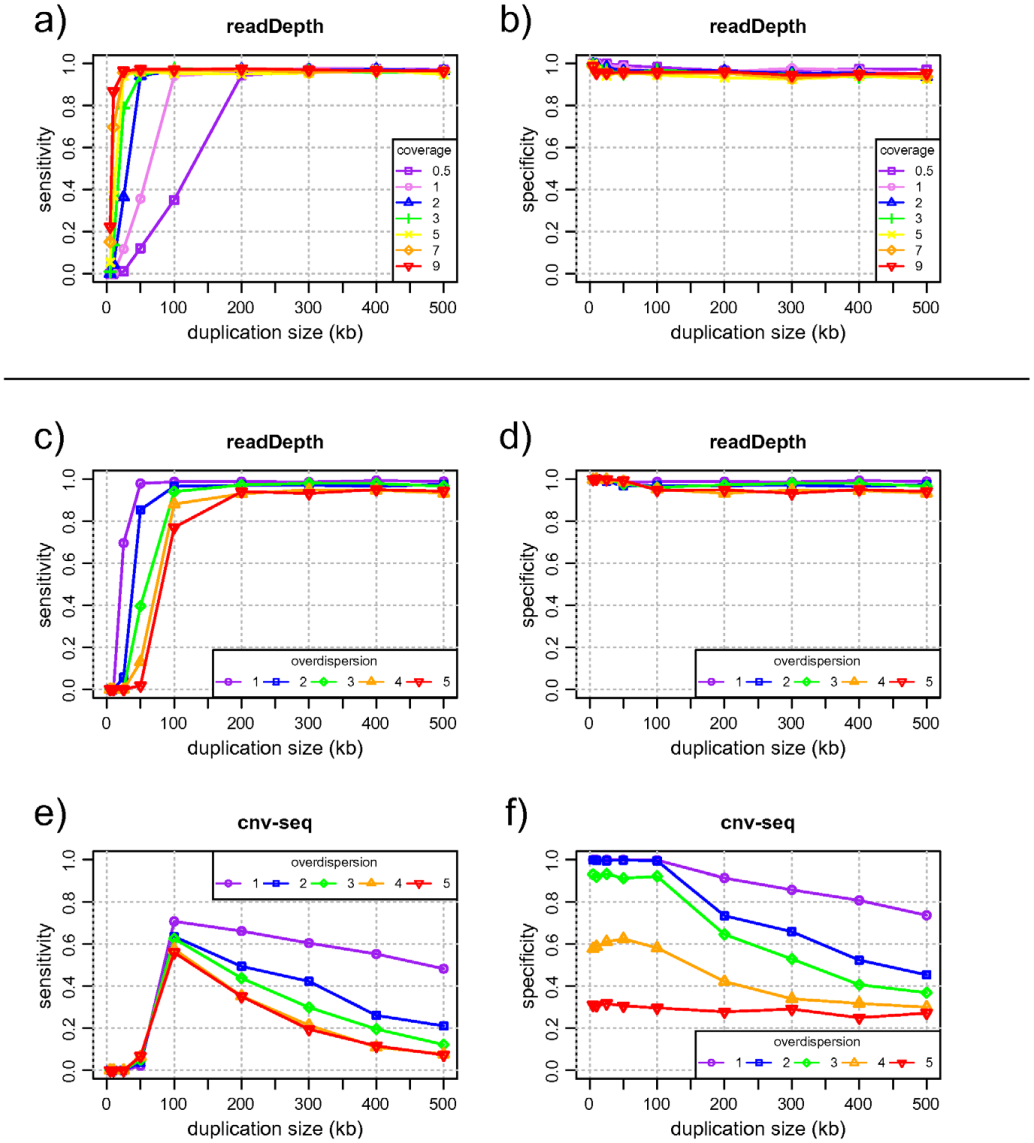
Algorithmic performance assessed on simulated data. a) readDepth sensitively detects small copy number alterations even at very low levels of sequence coverage. As additional reads increase the coverage, the algorithm is able to detect smaller alterations. b) Controlling the false discovery rate keeps the number of false positives very low. c/d) the readDepth algorithm is applied to simulated data with 1x coverage. When the read distribution is overdispersed, readDepth uses larger bins, effectively trading accuracy for resolution. e/f) When the cnv-seq algorithm is applied to the same data set, it tends to call many false breakpoints, resulting in lowered sensitivity and specificity. This problem is exacerbated by overdispersed data.

We show that readDepth detects CNAs with high sensitivity and specificity, even at low levels of genomic coverage. With a single lane of 76 bp Illumina paired-end sequencing that provides 0.5x coverage, readDepth reliably detects alterations that exceed 200 kbp in size. (Figure 2a,b). As the genomic coverage, and thus number of reads, increases, the algorithm can detect increasingly smaller aberrations while maintaining very high specificity.

We then compared the performance of readDepth to cnv-seq, which is another R package for detecting CNAs from sequencing data [12]. Since cnv-seq requires a matched normal sample, we followed the same random generation procedure as above to generate a reference sample with no CNAs. When the tools' performance is compared on the same generated data set, readDepth shows considerably higher sensitivity and specificity, even on data that is not overdispersed (Figure 2c–f). Examination of the results shows that this is largely due to cnv-seq misclassifying windows within a copy number alteration as normal. This results in hypersegmentation and inaccurate boundary calls. We also note that as the data deviates farther from a Poisson distribution, cnv-seq suffers from lower sensitivity and specificity. In contrast, readDepth adjusts for this overdispersion and maintains high sensitivity and specificity by sacrificing a small amount of resolution.

### Application - MCF-7 genome and breakpoint refinement

**Figure 3:**
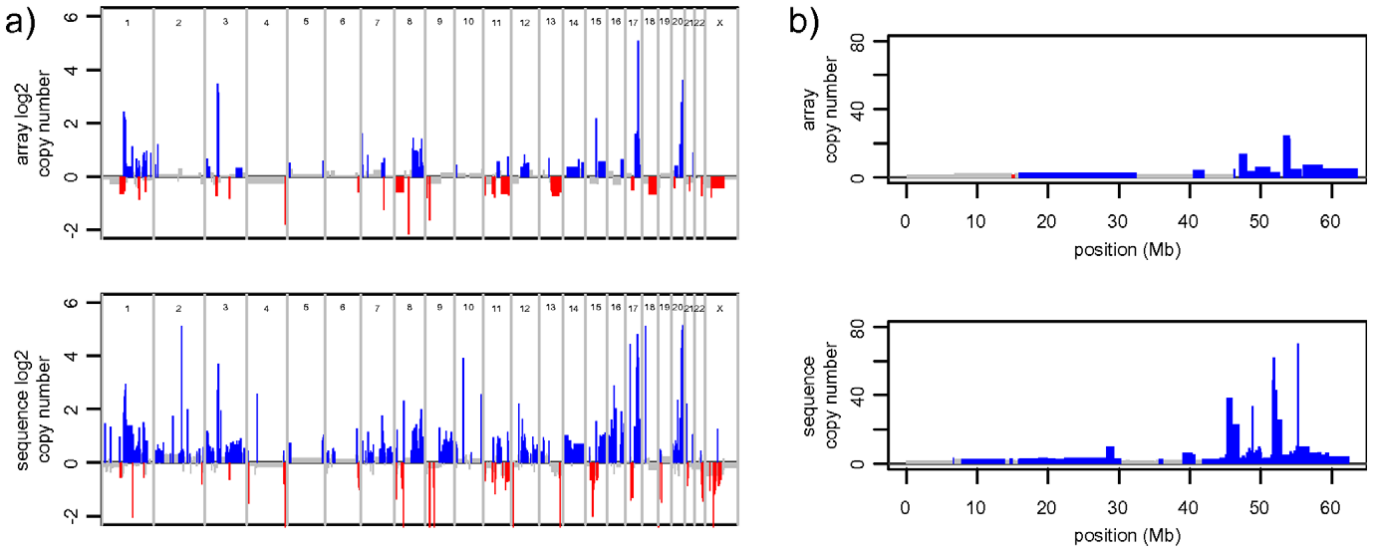
Comparison of array CN calls to sequence-based calls in MCF-7. a) a log_2_ plot of copy number alterations found in the MCF-7 breast cancer cell line. Sequence based copy number calls made with the readDepth package (bottom) reveal the same gross morphology seen by an assay done with a 100 k SNP array (top) b) an absolute copy number plot of MCF-7 chromosome 20, showing high level amplifications and fine-scale copy number changes not detectible with array-based methods.

To further test readDepth, we analyzed over 47 million uniquely mapping reads from the MCF-7 breast cancer cell line generated as 55 bp mate-pairs with the Illumina GAII sequencer. This represents 0.85-fold coverage of the reference genome. ReadDepth was applied to this data using an FDR rate of 0.01, which resulted in bins with a size of 25.3 kbp. As shown in Figure 3, the algorithm detects mostly the same gross regions of copy number change as aCGH on the Affymetrix 100 k SNP platform, albeit with better resolution and higher dynamic range. Two notable exceptions are large aberrations on chromosomes 2 and 9, which we believe to be biological differences between different sublines and/or passages of MCF-7. Manual examination of the raw data from these regions agrees with this conclusion. As expected, we find over 30% of the genome with altered copy number, and find almost eight times as many amplifications as deletions. The amplifications we find tend to be smaller, with a median size of 582 kbp, vs 942 kbp for deletions.

We then integrated breakpoint information derived from the mate-pair reads with information about read depth in order to more accurately delineate the boundaries of copy number alterations. Genomic breakpoints were detected from mate-pair data using a custom pipeline and the locations of these breakpoints were used as input to readDepth. After identifying segments of gain and loss, the algorithm adjusted segment boundaries if an unambiguous single breakpoint was located less than half of the bin size away from the segment boundary. This step resulted in the refinement of 99 breakpoints, effectively increasing the mean resolution of those copy number boundaries to 3.396 kbp, which is far lower than the 25.3 kbp resolution possible through the use of bins.

### Application - H1 genome using bisulfite sequencing reads

The use of bisulfite reads presents some unique challenges, as prior to sequencing, they are treated such that all non-methylated cytosines are converted to uracils, which are then read out of the sequencer as thymine bases [20]. We started with over 1.5 billion such reads from the H1 embryonic stem cell line generated on the Illumina GAII sequencer [21]. We then mapped these reads to the reference genome using Pash 3.0 [22] and created a comprehensive methylation map, where each cytosine base in the reference genome is annotated with the number of times methylation is observed at that position. Our package combines this methylation map with bisulfite mapability tracks, allowing us to accurately correct for GC-content bias.

With reads representing approximately 37-fold coverage of the genome, the algorithm is able to use a window size of 500 bp, resulting in a very high resolution picture of the CNAs in this cell line. We detect 6,722 copy number variants, mostly small, with 39% smaller than 10 kbp, and 80% smaller than 100 kbp. The number of deletions is over ten-fold higher, with 91% losses and 9% gains, but they tend to be smaller, with a median size of 17.5 kbp, compared to 45 kbp for amplifications. Chromosome 12 is especially aberrant, with most of the chromosome amplified (Figure S2). Since we have no array data to draw comparisons with, we used a high-resolution subset of the Database of Genomic Variants (version 9) [23]. We find that 71% of these amplifications and 49% of the losses overlap with known CNVs.

### Parallel processing

Through the use of the multicore and foreach R packages, readDepth splits the most CPU-intensive parts of the calculations among multiple processors [24]. We assessed runtime on a machine with 8 Intel Xeon CPUs running at 2.67 GHz. Using a simulated data set with 5-fold coverage of the genome, cnv-seq took 1651s to produce cnv calls, while readDepth was able to produce calls in 231s; a greater than 7-fold improvement.

## Methods

### Bin size determination

Given *n* reads from a genome of size *g*, and a bin size *b*, the mean number of reads per bin, λ, can be calculated as: λ = n*b/*g*. A Poisson model assumes that both the mean and the variance of the distribution are equal to λ. Since this is not the case in Illumina data, we model an overdispersed Poisson distribution by using a negative binomial distribution. The mean of the negative binomial distribution is also λ, but the variance can be controlled independently of the mean using a second parameter, *r*. By changing the value of *r*, we can choose an appropriate variance/mean ratio (VMR).

We generate the distributions using the rnbinom function in R, setting µ = λ, and the size parameter  =  λ/(d-1), where d is the input VMR. We then generate distributions using the expected number of reads with copy number of one, two, and three, and choose a threshold value for gains and losses that minimizes the number of bins that are misclassified. The FDR rate can then be calculated as the number of misclassified bins divided by the total number of bins. We progressively test lower bin sizes until we reach the smallest size possible under the constraints of the given false-discovery rate.

### Mapability correction

We developed mapability tracks by aligning all genomic sequences of a given length back to the human reference genome (hg18) with BWA [25]. The positions of all uniquely mapping reads were retained. To correct for mapability, the number of reads in a given bin was multiplied by the inverse of the percent mapability in that region. Regions with extremely low mapability (<25%) were filtered out to prevent overcorrection.

For bisulfite-mapability tracks, the same process was followed for read generation, then all cytosines were replaced with thymines to mimic the effects of bisulfite treatment. These were aligned to the reference genome using Pash 3.0, and read depth correction was carried out as above.

### GC-content correction

To correct for sequencing biases that arise due to preferential sequencing of certain levels of GC content, we normalize the read depth based on the GC content of each bin. These values incorporate mapability information, such that we only consider the GC content of mappable bases. To correct the data, we first calculate the average read depth for bins with GC content in intervals of 0.1%. We then use the LOESS method to fit a regression line to this data. The correction value for each bin is equal to the difference between the median read depth and the average read depth of that bin. We then scale the values such that the correction is neutral with respect to the total number of reads.

For bisulfite reads, we generate a methylation report for each cytosine in the genome using a pipeline built around Pash 3.0 [22]. These values are used to adjust the GC-content of each bin to reflect the status of both bases transformed by bisulfite treatment and bases protected by methylation. LOESS correction is then performed as described above.

### Segmentation

Segmentation is performed using circular binary segmentation, as implemented in the R package DNAcopy [19]. We use the default alpha value of 0.01 and for low-coverage data (MCF-7), we use a min.width value of 2. To reduce the number of false-positives in high-coverage data (H1), we use a min.width value of 4. This requirement of multiple consecutive bins significantly decreases the false discovery rate.

### Array Processing

MCF-7 copy number data assayed with the Affymetrix 100 k chip (50k_Xba240 and 50k_Hind240) was obtained from http://pevsnerlab.kennedykrieger.org/text/Affy_100K_Sample_Tumor_CN.txt. The data was segmented using circular binary segmentation with default values. Segments were called gains or losses if their mean value exceeded 1.5 standard deviations from the mean probe value.

### Data Availability

MCF-7 sequence reads are available at http://www.genboree.org/breastCellLineReads/ The 4 kb and 6 kb mate-pair reads were used in this analysis. H1 bisulfite sequencing data is available from public repositories as described in Lister, et al [20]. Copy number results for both cell lines are attached as Dataset S1 and Dataset S2.

## Discussion

The readDepth package is under active development and we anticipate adding a number of additional features in the future. Functions that will allow better visualization of results are currently being developed, as well as methods for detecting overdispersion automatically, rather than requiring it as an input parameter. We are also exploring methods for increasing the resolution of breakpoint calls when deep-sequencing data is available.

We expect that readDepth will be useful in a variety of different scenarios. Projects that produce low-coverage paired end sequencing can benefit from its ability to integrate breakpoint information for accurate boundary calls. Deep-sequencing projects will be able to leverage the multiple cores present in modern computers to detect CNAs quickly, even when analyzing billions of reads. Additionally, readDepth's first-of-its-kind ability to correct for GC-biases specific to bisulfite sequencing mean that it is uniquely well-suited to dealing with data coming from the burgeoning field of epigenomics.

The readDepth package is under active development and we anticipate adding a number of additional features in the future. Functions that will allow better visualization of results are currently being developed, as well as methods for detecting overdispersion automatically, rather than requiring it as an input parameter. We are also exploring methods for increasing the resolution of breakpoint calls when deep-sequencing data is available.

We expect that readDepth will be useful in a variety of different scenarios. Projects that produce low-coverage paired end sequencing can benefit from its ability to integrate breakpoint information for accurate boundary calls. Deep-sequencing projects will be able to leverage the multiple cores present in modern computers to detect CNAs quickly, even when analyzing billions of reads. Additionally, readDepth's first-of-its-kind ability to correct for GC-biases specific to bisulfite sequencing mean that it is uniquely well-suited to dealing with data coming from the burgeoning field of epigenomics.

The readDepth package runs on Linux and MacOSX, is released under the Apache 2.0 license, and is available at http://code.google.com/p/readdepth.

## Supporting Information

Figure S1 **Loess normalization.** The yoruban genome was binned and mapability corrected as described in the main text. Bins were then grouped by GC-content percentage in 0.01% increments, and the mean number of reads was calculated. The data shows considerable bias at extreme values of GC-content (top). Loess correction removes most of this bias (bottom).
(TIF)

Figure S2 **H1 cell line copy number calls.** A log_2_ plot of copy number alterations found in the H1 cell line. Though most CNAs are small, we find largescale amplification of chromosome 12.
(TIF)

Dataset S1 **MCF-7 copy number results.** Segmented copy number calls for the MCF-7 cell line.
(DAT)

Dataset S2 **H1 copy number results.** Segmented copy number calls for the H1 cell line.
(DAT)
