# Supplementary figures and images for: ReadDepth: A Parallel R Package for Detecting Copy Number Alterations from Short Sequencing Reads
Source: PLoS One. 2011 Jan 31;6(1):e16327. doi: 10.1371/journal.pone.0016327 (PMC3031566; doi:10.1371/journal.pone.0016327)

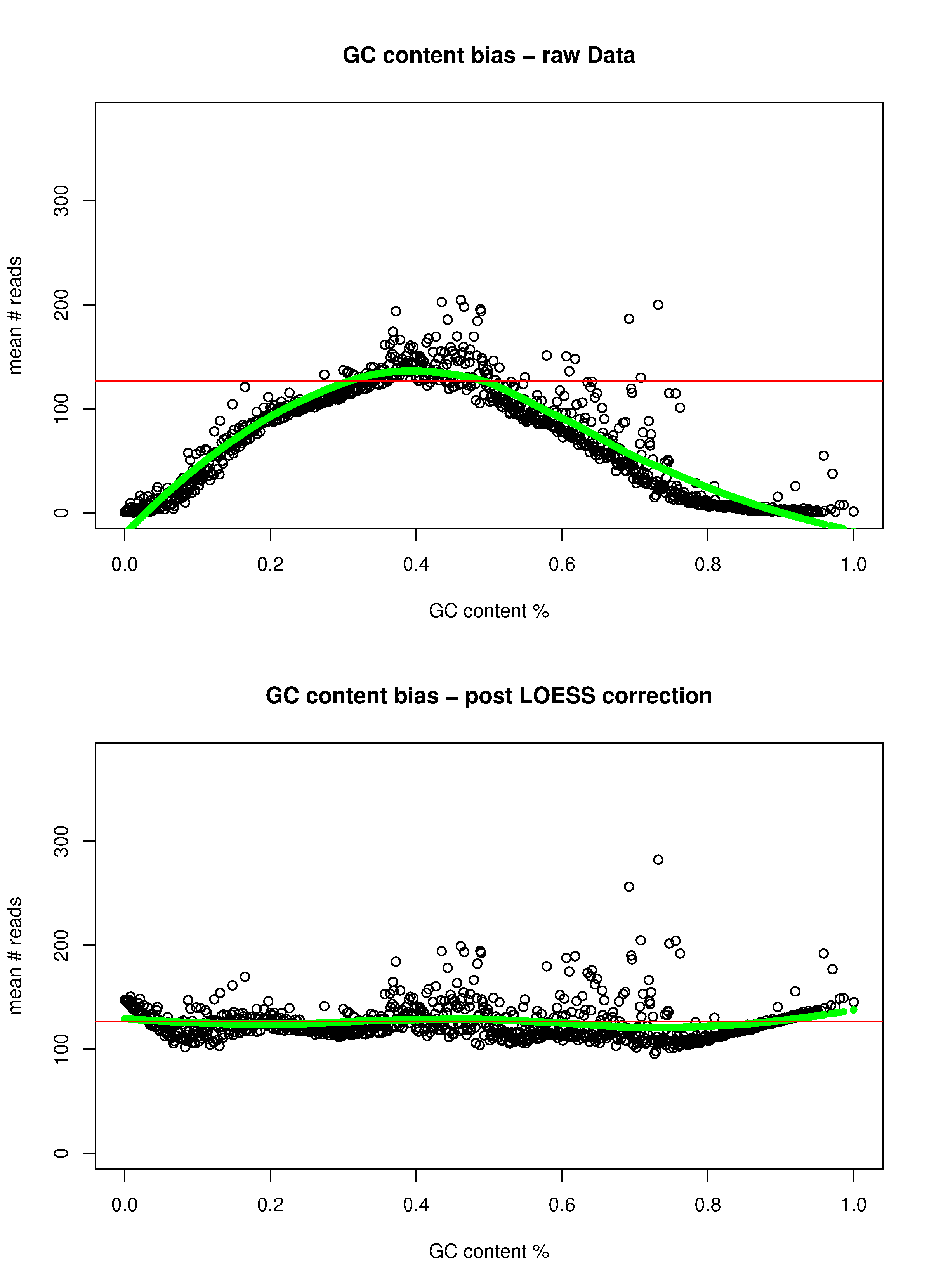

Supplement: Figure S1 — Loess normalization. The yoruban genome was binned and mapability corrected as described in the main text. Bins were then grouped by GC-content percentage in 0.01% increments, and the mean number of reads was calculated. The data shows considerable bias at extreme values of GC-content (top). Loess correction removes most of this bias (bottom). (TIF) [file pone.0016327.s001.tif]

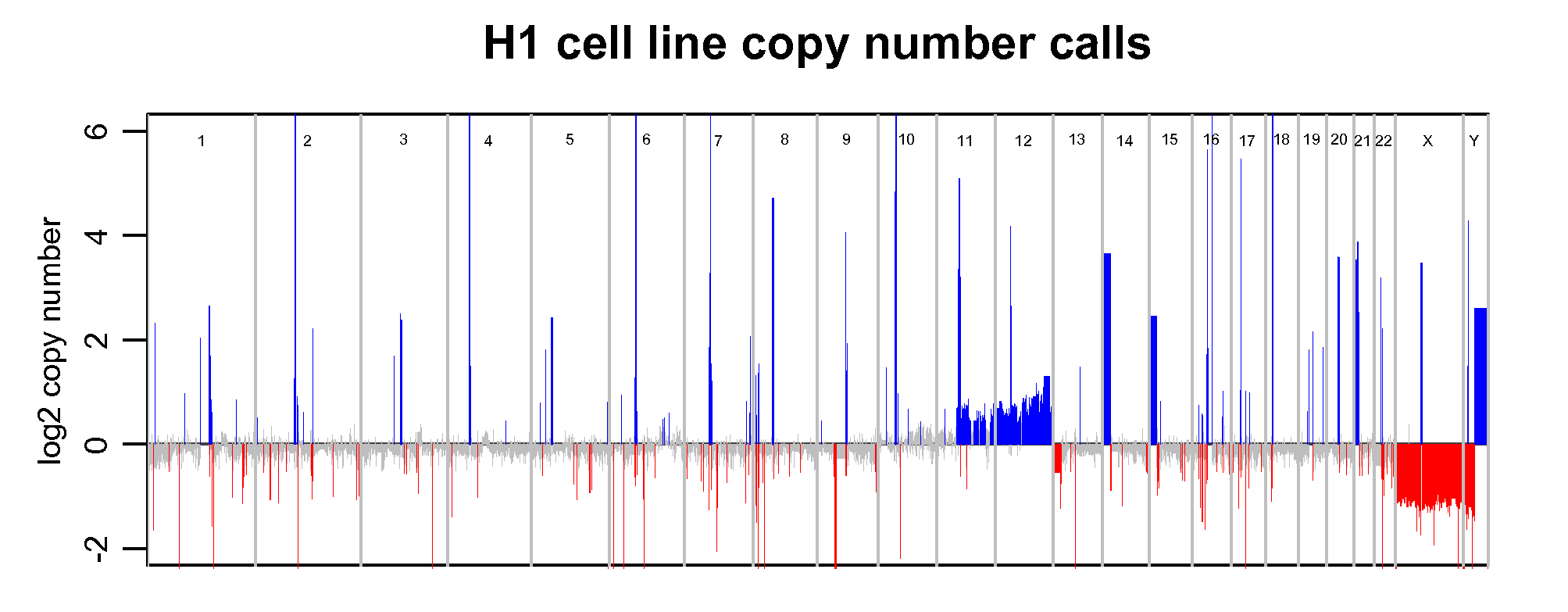

Supplement: Figure S2 — H1 cell line copy number calls. A log2 plot of copy number alterations found in the H1 cell line. Though most CNAs are small, we find largescale amplification of chromosome 12. (TIF) [file pone.0016327.s002.tif]
